# Mechanical CPR: Who? When? How?

**DOI:** 10.1186/s13054-018-2059-0

**Published:** 2018-05-29

**Authors:** Kurtis Poole, Keith Couper, Michael A. Smyth, Joyce Yeung, Gavin D. Perkins

**Affiliations:** 1 Warwick Medical School, University of Warwick, Coventry, UK; 2 South Central Ambulance Service NHS Foundation Trust, Bicester, UK; 3 University Hospitals Birmingham NHS Foundation Trust, Birmingham, UK; 4 West Midlands Ambulance Service NHS Foundation Trust, Brierly Hill, UK

**Keywords:** Cardiac arrest, Cardiopulmonary resuscitation, Mechanical CPR, Review

## Abstract

In cardiac arrest, high quality cardiopulmonary resuscitation (CPR) is a key determinant of patient survival. However, delivery of effective chest compressions is often inconsistent, subject to fatigue and practically challenging.

Mechanical CPR devices provide an automated way to deliver high-quality CPR. However, large randomised controlled trials of the routine use of mechanical devices in the out-of-hospital setting have found no evidence of improved patient outcome in patients treated with mechanical CPR, compared with manual CPR. The limited data on use during in-hospital cardiac arrest provides preliminary data supporting use of mechanical devices, but this needs to be robustly tested in randomised controlled trials.

In situations where high-quality manual chest compressions cannot be safely delivered, the use of a mechanical device may be a reasonable clinical approach. Examples of such situations include ambulance transportation, primary percutaneous coronary intervention, as a bridge to extracorporeal CPR and to facilitate uncontrolled organ donation after circulatory death.

The precise time point during a cardiac arrest at which to deploy a mechanical device is uncertain, particularly in patients presenting in a shockable rhythm. The deployment process requires interruptions in chest compression, which may be harmful if the pause is prolonged. It is recommended that use of mechanical devices should occur only in systems where quality assurance mechanisms are in place to monitor and manage pauses associated with deployment.

In summary, mechanical CPR devices may provide a useful adjunct to standard treatment in specific situations, but current evidence does not support their routine use.

## Background

High-quality chest compressions are a critical component in the cardiac arrest chain of survival [1]. Despite its importance, the sustained delivery of high-quality cardiopulmonary resuscitation (CPR) is infrequently achieved in clinical practice [2, 3].

Mechanical chest compression devices deliver high-quality external chest compressions, in place of a human rescuer. A number of devices are currently marketed, but devices can be broadly categorised as load distributing band or piston devices, based on the mechanism that is used to deliver compressions. The Autopulse (Zoll Medical, Chelmsford, MA, USA) is a load distributing band device, which consists of a large backplate that is positioned behind the patient and a band that encircles the patient’s chest to deliver compressions at a rate of 80 per minute and a depth of 20% of the anterior-posterior chest height. The LUCAS (Physio-Control Inc./Jolife AB, Lund, Sweden) is an example of a piston device, which also incorporates a mechanism for active chest recoil. It consists of two parts (a backplate and the piston mechanism), which link together to encircle the patient. The device consistently delivers compressions at a rate of 102 per minute and a depth of 5.3 cm in patients with a sternal height greater than 18.5 cm. The key theoretical benefit to the use of such devices is their ability to consistently deliver high-quality chest compressions, which has been associated with improved intra-arrest haemodynamic profiles [4, 5].

The purpose of this review is to provide an update on mechanical device use for both out-of-hospital cardiac arrest (OHCA) and in-hospital cardiac arrest (IHCA), an overview on device use in special circumstances, and guidance on deployment in the clinical setting.

### The importance of high quality CPR

International guidelines highlight the importance of high-quality chest compressions, which are defined as compressions at a depth of 5–6 cm and a rate of 100–120 per minute, allowing full chest recoil between compressions, and minimisation of interruptions [6, 7].

Despite consistent observational data showing the association between CPR quality and patient outcome [8, 9], the delivery of high-quality manual chest compressions is challenging in both the out-of-hospital and in-hospital settings [2, 3]. Specific barriers include provider fatigue [10, 11], physical effort to overcome stiffness of the patient’s thoracic cage [12], and compressible underlying surfaces, such as mattresses, which can lead to shallow chest compressions [13, 14]. For example, in an analysis of 9136 OHCA patients, only 45% received the recommended guideline chest compression depth [15].

In contrast to manual chest compressions, mechanical devices are not subject to the physical limitations of the rescuer and are able to consistently deliver high-quality chest compressions.

### Current treatment recommendations

In 2015, the International Liaison Committee on Resuscitation’s (ILCOR) consensus on science and treatment recommendation process evaluated the use of mechanical chest compression devices in clinical practice [16]. The evidence evaluation process made a single treatment recommendation to cover all settings and all mechanical device types.

Based on the expert review of the available data, ILCOR made a weak recommendation (moderate quality evidence) against the routine use of mechanical devices in clinical practice. However, the review acknowledged situations where the delivery of high quality manual chest compressions may be impractical or dangerous to rescuers. In such circumstances, ILCOR made a weak recommendation based on low-quality evidence supporting the use of mechanical devices. The commentary accompanying the treatment recommendation highlighted concerns that the deployment of mechanical devices without appropriate training might cause patient harm through an increase in no-flow time during the early part of the cardiac arrest and delay defibrillation in patients with a shockable rhythm.

### Out-of-hospital cardiac arrest

#### Routine deployment in OHCA

The routine deployment of mechanical devices has been robustly tested in the pre-hospital setting in large high-quality randomised controlled trials. In 2014–2015, the CIRC (Circulation Improving Resuscitation Care) [17], LINC (LUCAS in Cardiac Arrest) [18], and PARAMEDIC (Prehospital Randomised Assessment of a Mechanical Chest Compression Device in Cardiac Arrest) [19] trials were published. These studies, alongside two earlier small randomised studies [20, 21], are summarised in Table 1.

**Table 1:** Summary of randomized controlled trials comparing routine use of mechanical CPR with manual CPR in OHCA

	Load-distributing band trials		Piston-device trials		
	Hallstrom et al. [20]^a^	CIRC [17]	Smekal et al. [21]	LINC [18]	PARAMEDIC [19]
Design	Efficacy superiortity multicentre RCT	Efficacy equivalence multicentre RCT	Pilot multicentre RCT	Efficacy superiority multicentre RCT	Effectiveness superiority multicentre RCT
Randomisation	Cluster- EMS station(s) (ratio 1:1)	Patient (ratio 1:1)	Patient (ratio 1:1)	Patient (ratio 1:1)	Cluster- ambulance (ratio 2 manual:1 mechanical)
Inclusion criteria	Adult non-traumatic OHCA	Adult OHCA of cardiac aetiology. EMS arrival time ≤ 16 min	Adult non-traumatic OHCA	Adult unexpected non-traumatic OHCA where resuscitation was appropriate	Adult non-traumatic OHCA where a trial vehicle was first ambulance on scene
Number of cases analysed	1071 (1071 randomised)	4231 (4753 randomised)	149	2589 (2593 randomised)	4470 (4471 randomised)
Setting	US/Canada	US /Europe	Sweden	Europe	UK
Sponsor	Industry	Industry	Academic^b^	Industry	Academic
Device used	Autopulse	Autopulse	LUCAS	LUCAS	LUCAS
Primary outcome	4-h survival: Manual 29.5% vs mechanical 28.5%, *p* = 0.74	STD Manual 11.0% vs mechanical 9.4%, adj. OR 1.06 (95% CI 0.83, 1.37)^c^	Not specified	4-h survival Manual 23.7% vs mechanical 23.6%, treatment difference − 0.05 (95% CI − 3.3, 3.2)	30-day survival Manual 7% vs mechanical 6%, adj. OR 0.86 (95% CI 0.64, 1.15)
Key secondary outcomes	Cardiac aetiology group (*n* = 767): STD: Manual 9.9% vs mechanical 5.8%, *p* = 0.06 (adjusted) Good neurological outcome: Manual 7.5% vs mechanical 3.1%, *p* = 0.006	Sustained ROSC: Manual 32.3% vs mechanical 28.6%, adj. OR 0.84 (95% CI 0.73, 0.96) Good neurological outcome in survivors: Manual 48.1% vs mechanical 44.4%, adj. OR 0.80 (95% CI 0.47, 1.37)	ROSC: Manual 32% vs mechanical 41%, *p* = 0.30 STD: Manual 10% vs mechanical 8%, *p* = 0.78	STD: Manual 9.2% vs mechanical 9.0%, treatment difference − 0.15 (95% CI − 2.4, 2.1) Good neurological outcome: Manual 7.3% vs mechanical 8.1%, treatment difference 0.78 (95% CI − 1.3, 2.8)	ROSC: Manual 31% vs mechanical 32%, adj. OR 0.99 (95% CI 0.86, 1.14) Good neurological outcome: Manual 6% vs mechanical 5%, adj. OR 0.72 (95% CI 0.52, 0.99)

^a^Trial stopped early by data monitoring board

^b^One author received consulting fee from device manufacturer

^c^Primary outcome result within pre-specified boundary of equivalence. Trial stopped early in accordance with pre-specified stopping rule

*EMS* Emerency Medical Service, *OHCA* out-of-hospital cardiac arrest, *RCT* randomised controlled trial, *STD* survival to discharge, *adj OR* adjusted odds ratio

The CIRC trial was an industry sponsored trial, designed to determine equivalence, superiority, or inferiority in survival to hospital discharge for OHCA patients who were randomised in a 1:1 ratio to receive either manual CPR or Autopulse CPR [17]. The study was terminated early, in accordance with pre-defined stopping rules, after 4753 randomised patients were enrolled. Of those randomised, 4231 were included in the intention-to-treat analysis. Overall, manual CPR showed a numeric increase in survival to hospital discharge compared to Autopulse CPR (11.0 versus 9.4%). The adjusted odds ratio (OR) was 1.06 (95% confidence interval (CI) 0.83, 1.37) after adjustment for covariates and interim analyses. This fell within the pre-defined equivalence region (OR 0.69–1.44), although the width of the equivalence margin incorporates the potential for both significant harm and benefit [22]. Overall hospital survival rate was higher than that reported in similar studies (PARAMEDIC 30 day survival 6.6%; LINC hospital survival 8.0%). This may reflect the stringent study inclusion criteria and intensive training and oversight by the study team which emphasised the importance of high-quality CPR.

The LINC trial was also an industry-sponsored efficacy trial, in which OHCA patients were randomised in a 1:1 ratio to receive either LUCAS or manual CPR [18]. The trial used a modified treatment algorithm for the LUCAS arm that incorporated defibrillation without rhythm assessment and 3-minute periods between rhythm assessments. The study randomised 2593 patients, of which 1589 were included in the intention-to-treat analysis. In relation to the primary outcome of 4-h survival, LUCAS was not superior to manual chest compressions (treatment difference 0.05%, 95% CI − 3.3, 3.2).

The PARAMEDIC study was an academic pragmatic cluster randomised trial, in which ambulance vehicles were randomised in a 2:1 ratio to receive either manual CPR or LUCAS CPR. The treatment allocation of the individual patient was determined by the first vehicle to arrive on scene. The study included 4471 patients, of which 4470 were included in the primary analysis. In relation to the primary outcome of 30-day survival, LUCAS was not superior to manual compressions (adjusted OR 0.86, 95% CI 0.64, 1.15). The study experienced a high incidence of non-compliance in the LUCAS CPR arm, such that only 60% received mechanical CPR. A CACE (compiler average causal effect) analysis, which accounts for non-compliance, generated similar findings to the main analysis [23].

The PARAMEDIC study also collected cost-effectiveness and quality of life data following hospital discharge [24–26]. The long-term (up to 12 months) outcome analysis found no clinically important differences between groups in relation to outcomes such as survival, neurological outcome, and quality of life at 3 months and 12 months, although the analysis was subject to a high risk of attrition bias [24]. The cost-effectiveness analysis found that routine use of mechanical CPR devices in the out-of-hospital setting was not cost-effective [25].

A systematic review and meta-analysis by Gates et al. [22] incorporated all five randomised controlled trials, as summarised in Table 1, with a total patient population of 12,206. The random-effects meta-analysis found that mechanical CPR was not superior to manual CPR, in relation to key outcomes such as return of spontaneous circulation (OR 0.96, 95% CI 0.85, 1.10), survival at hospital discharge/30 days (OR 0.89, 95% CI 0.77, 1.02), or good neurological outcome (OR 0.76, 95% CI 0.53, 1.11).

In summary, these data do not support the routine use of mechanical CPR in OHCA.

#### Use during transfer to hospital

The Universal Termination of Resuscitation rule guides clinical teams when to consider transport from scene of the cardiac arrest to hospital with on-going CPR [27]. Other indications for transport include situations where potentially lifesaving treatments cannot be delivered outside a hospital, such as extra-corporeal CPR, re-warming after hypothermic cardiac arrest, and invasive procedures (e.g. primary percutaneous coronary intervention (pPCI)) [28]. The process of intra-arrest transport typically requires two phases: extrication of the patient to the ambulance and vehicular transfer to the hospital. In each of these phases, a key challenge for the EMS crew is the safe ongoing delivery of high-quality CPR.

The majority of OHCAs occur in the patient’s home [29]. As such, a key challenge in the extrication stage is manoeuvring past obstacles and downstairs whilst continuing to deliver CPR. In an observational study, researchers analysed the pauses associated with this process prior to and following the introduction of mechanical CPR to facilitate the extrication process [30]. In the first period where manual CPR was provided, the median chest compression pause during extrication was 270 s (interquartile range (IQR) 201, 387), with some pauses recorded as being in excess of 10 min. In contrast, after introduction of mechanical CPR, chest compressions were delivered continuously during extrication, except for the pause required to deploy the mechanical device (median 39 s (IQR 29, 47).

For the vehicular transfer to hospital stage, there are three main concerns. Firstly, delivery of manual CPR in a moving vehicle is inherently unsafe, and exposes both the patient and EMS provider to risk of injury or death [31, 32]. Secondly, there is a risk of suboptimal CPR delivery due to acceleration forces during ambulance transport [33]. However, evidence from clinical studies has been mixed with some reporting a similar quality of manual chest compressions prior to and during transfer, whilst other studies have reported either a transfer related deterioration or increased variability in quality [30, 34–36]. Finally space consideration, such as in the context of transport by helicopter, may render delivery of manual CPR difficult or impossible. In this setting, mechanical chest compression devices have been used to effectively deliver ongoing CPR during transport [37].

Based on these data, in particular the safety concerns associated with the delivery of manual CPR during transport, it would seem reasonable to consider the use of mechanical CPR during transport to hospital.

#### Use in the emergency department

The emergency department (ED) cardiac arrest population comprises both OHCA patients that did have a pre-hospital ROSC and patients that arrest in the emergency department. In view of limited personnel and a case-mix that likely includes prolonged cardiac arrests, mechanical device use in the ED may appear an attractive solution. A recent multi-centre Japanese observational study analysed the outcome of 6537 cardiac arrest patients (5619 manual CPR, 918 mechanical CPR) treated in the ED [38]. The use of a device was associated with reduced likelihood of ROSC (unadjusted OR 0.90, 95% CI 0.77, 1.06; adjusted 0.71, 95% CI 0.53, 0.94) and hospital survival (unadjusted OR 0.97, 95% CI 0.62, 1.51; adjusted 0.40, 95% CI 0.20, 0.78). However, the decision as to whether or not to use a mechanical device was made on a patient by patient basis, such that there is a high risk that selective enrolment introduced unmeasured confounding variables which may have biased the results.

In a before–after study in two Singaporean hospitals, researchers compared patient outcomes prior to and following the implementation of the Autopulse device as part of the treatment for ED cardiac arrests [39]. In total, 1011 (459 manual CPR period; 552 mechanical CPR period) patients were studied. Unadjusted ORs show an association between treatment in the mechanical CPR period and improved ROSC (OR 1.89, 95% CI 1.43, 2.50), hospital survival (OR 2.55, 95% CI 1.00, 6.47), and good neurological outcome (OR 8.7, 95% CI 1.1, 71.6), but the interpretation of these findings is complicated by marked differences in baseline patient characteristics (e.g. initial rhythm, arrest location). Adjusted analyses showed an association between treatment in the mechanical CPR period and ROSC (OR 1.60, 95% 1.16, 2.22), but no association was observed in relation to any other outcome.

The reason for the apparent contrast in findings may reflect differences in patient population, study risk of bias (selection bias, effect of unmeasured confounders), or the strategy used to deploy the mechanical device. In particular, the team that deployed the device in Ong et al.’s study [40] had received focussed team training to optimise device deployment, thereby minimising pauses associated with its use. Overall, the findings of these studies with their inherent risk of bias do not support the routine use of mechanical CPR in the ED.

### In-hospital cardiac arrest

In contrast to the OHCA setting, few studies have sought to evaluate the routine use of mechanical CPR in the IHCA setting. A recent systematic review and meta-analysis identified only three randomised controlled trials which enrolled 234 patients [41].

The largest of these trials, and the only study published in the last 20 years, enrolled 150 in-hospital cardiac arrest patients who were randomised to receive either mechanical CPR delivered by a piston device or manual CPR [42]. The study report is available only in Chinese. After translation, unfortunately, key patient characteristics, such as initial rhythm, are not reported. The study reported that the use of a mechanical device improved survival to hospital discharge (OR 2.81, 95% CI 1.26, 6.24). This trial, alongside the two other trials [43, 44], are summarised in Table 2.

**Table 2:** Summary of randomized controlled trials comparing routine use of mechanical CPR with manual CPR in IHCA

	Load-distributing band trials	Piston-device trials	
	Halperin et al. [44]	Taylor et al. [43]	Lu et al. [42]^a^
Design	Efficacy superiortity single-centre RCT	Efficacy superiortity single-centre RCT	Efficacy superiority single-centre RCT
Randomisation	Patient (ratio 1:1)	Patient (ratio 1:1)	Patient (ratio 1:1)
Inclusion criteria	IHCA of less than 20-min duration following tracheal intubation and adrenaline administration	IHCA of less than 10-min duration	IHCA of less than 10-min duration
Number of cases analysed	34	50	150
Setting	US	US	China
Sponsor	Academic^b^	Academic	Unclear
Device used	Load-distributing band device	Thumper device	Thumper device
Primary outcome	ROSC Manual 18% vs mechanical 47%, OR 4.15 (95% CI 0.86, 19.92)	One-hour survival Manual 38% vs mechanical 42%, OR 1.14 (95% CI 0.37, 3.55)	STD Manual 15% vs mechanical 33%, OR 2.81 (95% CI 1.26, 6.24)
Key secondary outcomes	24-h survival: Manual 6% vs mechanical 18%	STD: Manual 8% vs mechanical 13%, OR 1.71 (95% CI 0.26, 11.26)	ROSC: Manual 38% vs mechanical 55%, OR 2.03 (95% CI 1.06, 3.90)

^a^Published only in Chinese

^b^Nine authors report equity interest in company holding device patent

*IHCA* in-hospital cardiac arrest, *RCT* randomised controlled trial, *STD* survival to discharge

The meta-analysis of the three randomised trials alongside six (455 patients) observational studies found very low quality evidence supporting an association between mechanical CPR use and increased likelihood of ROSC (OR 2.14, 95% CI 1.11, 4.13) and survival to hospital discharge/30 days (OR 2.34, 95% CI 1.42, 3.85) [41]. Neurological outcome was not assessed in any study. The results of the meta-analysis were broadly consistent between the sub-groups of randomised controlled trials and observational studies.

These findings seemingly contrast with research findings from out-of-hospital studies [22]. Reasons for this apparent discrepancy may reflect differences in either the quality of evidence or clinical characteristics between the two settings, such that mechanical devices may be more effective than manual chest compressions in the hospital setting. Examples of such characteristics include the opportunity for early device deployment and the challenges of delivering effective manual chest compressions on a bed mattress.

Based on this discrepancy, the need for a randomised controlled trial in the in-hospital setting was recently highlighted as a research priority [45]. The ongoing COMPRESS-RCT (ISRCTN38139840) study is assessing the feasibility of undertaking such a trial.

### Risk of injury during mechanical device use

Injuries secondary to manual chest compression are common and well-reported [46]. Common injuries include fractures (rib, sternal), pneumothoraces, and visceral organ damage (liver, spleen, heart) [46–48]. Several case reports have purportedly linked mechanical device use with clinically important injuries, thereby driving concern that mechanical devices may increase risk of injury compared with manual CPR [49–51]. Whilst evidence from cohort studies has produced mixed results, interpretation of these studies is challenging as they are prone to selection bias and the quality of manual CPR delivered, as the comparator group, is generally not recorded [52–55]. The PARAMEDIC, LINC, and CIRC trials were designed to examine the clinical effectiveness of mechanical devices, rather than to specifically examine injury, but it is noteworthy that these trials did not report a difference in injury patterns or severity between patients receiving manual and mechanical chest compression [17–19].

Koster et al. recently published a non-inferiority randomised controlled trial that provided the most robust evidence in relation to injury attributable to mechanical chest compression devices [56]. In total, 374 patients were randomised to receive LUCAS CPR, Autopulse CPR, or to continue to receive manual CPR [56]. The primary outcome was serious or life-threatening resuscitation-related visceral organ damage. Outcome data were available for 90% of participants. Compared to manual CPR, the non-inferiority analysis showed that LUCAS did not increase the risk of injury. However, an increase in injury could not be ruled out with the Autopulse device. The depth of manual chest compressions delivered in the manual CPR arm was 48 mm (SD 9), which is slightly lower than the current recommended target depth of 50 mm [6].

### Adjunct to advanced treatments

#### pPCI and CT scan

Delivery of high quality manual chest compressions during imaging procedures, such as coronary angiography or CT scan, is practically challenging due to the required positioning of the radiology equipment. Several case series describe the experience of specialist centres in performing intra-arrest coronary angiography and pPCI facilitated by mechanical CPR, with reported hospital survival rates of approximately 25% [57–59]. Wagner et al. [58] acknowledge that movement during CPR increases the complexity of the procedure, but recommend strategies such as a brief CPR pause during stenting to overcome this challenge. Whether the routine transfer of patients in refractory cardiac arrest for pPCI during on-going CPR improves patient outcome remains to be determined.

Transporting a patient in cardiac arrest to the CT scanner is rarely likely to improve management. However, there may be cases where a patient scheduled for a CT scan has a cardiac arrest just prior to the commencement of the scan. In these circumstances, it may be reasonable to proceed with the scan to confirm the presence of a treatable reversible cause, such as a massive pulmonary embolism. In such patients, imaging of acceptable quality can be obtained whilst CPR is delivered by a mechanical device [60].

#### Extracorporeal CPR

Extracorporeal CPR (E-CPR) is a cardiac arrest treatment strategy whereby patients are placed on cardiopulmonary bypass. Whilst evidence supporting E-CPR is limited and based on observational studies, a number of regions have established systems where E-CPR may be offered to patients who might narrow inclusion criteria [61, 62]. Several of these systems use mechanical CPR to facilitate the insertion of the E-CPR intravascular cannulae [63–65].

In Paris, for example, mechanical CPR has been used as a bridge to pre-hospital E-CPR in 156 patients, with an overall ROSC rate of 77.8% [63]. Similarly, the Australian CHEER study included 26 patients in refractory cardiac arrest that were treated with a care protocol comprising mechanical CPR, therapeutic hypothermia, E-CPR, and pPCI [64]. Fourteen (54%) survived to hospital discharge, all of whom had full neurological recovery.

The ongoing Prague-based Hyperinvasive Approach in Cardiac Arrest trial (NCT01511666) will provide important new information about the role of mechanical CPR as a bridge to E-CPR [66].

#### Organ donation

Uncontrolled donation after circulatory death (uDCD) provides a system whereby organs can be retrieved after sudden cardiac arrest in cases where it has not been possible to obtain a ROSC [67]. This enables the retrieval of organs such as lungs, kidneys, and liver. Whilst this concept poses legal, ethical, and practical challenges, it provides an opportunity to increase the number of viable donor organs [67, 68].

The use of mechanical CPR as a bridge to non-heart beating donation has also been described in a number of countries [37, 67]. The use of mechanical CPR provides a system to limit warm ischaemic time [68] whilst potentially providing a controlled setting in which consent for donation can be sought. In Spain, a comparable number of organs were transplanted with a similar graft failure rate following implementation of mechanical CPR as part of a uDCD protocol [69]. The study also highlighted the challenge of delivering such a system through its report that three patients, following protocol implementation and initiation of mechanical CPR, obtained ROSC, of which one made a good recovery.

Clinical decisions to refer for advanced lifesaving interventions (e.g. E-CPR) versus organ donation present ethical dilemmas that require careful consideration [70].

### Optimising clinical use of mechanical devices

#### Timing of deployment

In systems where mechanical devices are available, a key challenge for the clinician is the decision as to the time point during the cardiac arrest at which to deploy the mechanical chest compression device. In a meta-regression of out-of-hospital data, Bonnes et al. [71] identified an association between improved outcome and earlier device deployment.

A sub-group analysis in the PARAMEDIC trial identified decreased 30-day survival in patients treated with a mechanical device that presented in a shockable rhythm (odds ratio 0.71, 95% CI 0.52, 0.98) [19]. One plausible explanation for this is that the study protocol required deployment of the mechanical device prior to defibrillation, leading to delays in defibrillation in the mechanical CPR arm, although this delay was not measured in the trial. In contrast, the LINC study, which adopted a modified mechanical CPR treatment algorithm, found no difference in outcome between treatment groups in patients presenting in a shockable rhythm (e.g. hospital discharge treatment difference 0.6%, 95% CI − 5.6, 6.9), despite an increased median time to first shock in the mechanical CPR arm (mechanical 4 min (IQR 2,5) vs manual 3 min (IQR 2, 4), *P* < 0.001) [72].

Delivery of high-quality manual chest compressions for a prolonged period of time is physically exhausting [11, 73]. In the context of a prolonged cardiac arrest with limited personnel available, use of a mechanical chest compression device may be a reasonable strategy to avoid the potential harm associated with suboptimal chest compression delivery.

Based on these data, it would seem reasonable to deploy devices early in circumstances where high-quality manual chest compressions cannot be safely delivered. In patients where high-quality CPR is deliverable, delayed deployment would seem prudent in patients in a shockable rhythm.

#### Deployment

The key modifiable risk associated with mechanical device use is the pause associated with device deployment. There is a risk that prolonged pauses associated with device deployment during the early part of a cardiac arrest event may offset the subsequent potential benefit of improved CPR.

In clinical practice, published literature reports marked variability in the hands-off time during device deployment, with pauses in excess of 1 minute being reported [74]. In the LINC trial, the median reported chest compression pause associated with device deployment was 36.0 s (IQR 19.5, 45.5) [75]. However, subsequent improvement in flow-fraction following device deployment meant that the median flow-fraction over the first 10 minutes of the cardiac arrest was higher in the mechanical CPR arm (mechanical 0.84 (IQR 0.78, 0.91) vs manual 0.79 (IQR 0.70, 0.86), *p* < 0.001). A similar pattern was observed in the CIRC trial [17].

High-quality training that focuses on minimising pauses is an effective strategy to reduce chest compression pauses associated with device deployment [40, 76]. Levy et al. [76] implemented a system which incorporated a choreographed team approach to device deployment, debriefing, mock resuscitation drills, and adaptations to the deployment process to minimise pauses. The implementation of this system was associated with a significant reduction in the median pause immediately prior to the first mechanical chest compression (21 (IQR 15, 31) vs 7 (IQR 4, 12) s, *p* < 0.001). Whenever mechanical CPR systems are deployed, a careful system of quality assurance should be initiated to ensure optimal device deployment and avoidance of prolonged interruptions in chest compressions.

#### Future developments

The integration of mechanical CPR with other technologies, such as active compression–decompression technology or impedance threshold devices [77], has the potential to impact on the efficacy of currently marketed mechanical chest compression devices. However, a recent study found that the integration of active compression–decompression technology with a LUCAS mechanical chest compression device did not improve end tidal carbon dioxide, compared with use of a LUCAS without the technology [78].

There may be the opportunity in the future for mechanical chest compression devices to titrate chest compression delivery to physiological endpoints, such as end-tidal carbon dioxide or arterial blood pressure [79].

## Conclusions

The provision of high-quality CPR is a key modifiable factor associated with survival in cardiac arrest. Mechanical chest compression devices consistently deliver high-quality chest compressions, but this does not translate into improved patient outcomes when devices are routinely used in OHCA. Further trials are needed to evaluate the routine use of mechanical devices in IHCA.

The use of mechanical devices in specific circumstances (e.g. ambulance/helicopter transport, pPCI) where high-quality chest compressions cannot be safely delivered may be a reasonable strategy. In all situations where mechanical devices are used, clinicians must ensure that the device is deployed with minimal interruption to chest compression delivery.

## Abbreviations

CPR: Cardiopulmonary resuscitation
E-CPR: Extracorporeal cardiopulmonary resuscitation
ED: Emergency department
EMS: Emergency Medical Service
IHCA: In-hospital cardiac arrest
ILCOR: International Liaison Committee on Resuscitation
OHCA: Out-of-hospital cardiac arrest
pPCI: Primary percutaneous coronary intervention
ROSC: Return of spontaneous circulation
uDCD: Uncontrolled donation after circulatory death
